# Radiative coupling of two quantum emitters in arbitrary metallic nanostructures

**DOI:** 10.1038/s41598-022-10624-y

**Published:** 2022-04-27

**Authors:** JingFeng Liu, Gengyan Chen, Lingyan Li, Renming Liu, Wei Li, Guanghui Liu, Feng Wu, Yongzhu Chen

**Affiliations:** 1 College of Electronic Engineering (College of Artificial Intelligence), South China Agricultural University, Guangzhou, 510642 China; 2 School of Optoelectronic Engineering, Guangdong Polytechnic Normal University, Guangzhou, 510665 China; 3 School of Physics and Electronics, Henan University, Kaifeng, 475004 China; 4 State Key Laboratory of Optoelectronic Materials and Technologies, School of Physics, Sun Yat-Sen University, Guangzhou, 510275 China

**Keywords:** Nanophotonics and plasmonics, Quantum optics

## Abstract

We propose a general formalism beyond Weisskopf–Wigner approximation to efficiently calculate the coupling matrix element, evolution spectrum and population evolution of two quantum emitters in arbitrary metallic nanostructures. We demonstrate this formalism to investigate the radiative coupling and decay dynamics of two quantum emitters embedded in the two hot spots of three silver nano-spheroids. The vacuum Rabi oscillation in population evolution and the anti-crossing behavior in evolution spectrum show strong radiative coupling is realized in this metallic nanostructure despite its strong plasmon damping. Our formalism can serve as a flexible and efficient calculation tool to investigate the distant coherent interaction in a large variety of metallic nanostructures, and may be further developed to handle the cases for multiple quantum emitters and arbitrary dielectric–metallic hybrid nanostructures.

## Introduction

The coherent interaction between spatially separated quantum emitters, e.g., atoms, dye molecules and quantum dots, determines their decay dynamics and facilitates quantum information processing. In solid-state implementation, the photon-mediated radiative coupling between quantum emitters is a promising approach due to the low decoherence rate, high velocity and matured on-chip photonic technology^1^. The radiative coupling can be tailored by suitably designing the electromagnetic environment of the quantum emitters^2^. For instance, optical lenses^3^ and waveguides^4^ can collect and transfer the emitted photon from one quantum emitter to another one, while optical cavities^5^ can enhance the photon-mediated interaction, even realize the strong radiative coupling^6–11^. The strong radiative coupling and entanglement of two quantum emitters can occur over longer distance in the photonic crystal dimers^12–14^ and photonic band gap material^15,16^. Apparently, the crucial requirement for strong radiative coupling is a tight confinement of electromagnetic field.

Nevertheless, due to the diffraction limit, the light field in any dielectric nanostructure, e.g., optical cavities^5^, can only be confined down to the light wavelength scale. So the electric field at each quantum emitter and hence the radiative coupling between quantum emitters are both limited. To break this limit and further enhance the radiative coupling, metallic nanostructures are proposed as an alternative scheme. Metallic nanostructures, e.g., plasmonic cavities^17^, can squeeze light field into extremely small volume beyond diffraction limit and of the nanometer scale^18^, and produce enormously strong electric field (hot spots). By embedding quantum emitters separately into these hot spots, strong radiative coupling^19–21^ and entanglement^22^ can be realized. Furthermore, both the deep subwavelength confinement associated with surface plasmons and the one-dimensional character of plasmonic waveguides can be simultaneously exploited to enhance the interaction between distant quantum emitters, introducing energy transfer^23–25^, superradiance^23,26^ and entanglement^27,28^. Due to the rapid progress in nanofabrication and measurement techniques, various metallic nanostructures are elaborately designed and fabricated to realize diverse goal, e.g., a U-shaped gold nanostructure can realize selective excitation and detection of two coupled quantum emitters from the far field^29^. In metallic nanostructures, the dominant coupling mechanism between quantum emitters is virtual plasmon exchange, rather than direct radiative coupling^30,31^.

To investigate the plasmon-mediated radiative coupling between quantum emitters in metallic nanostructures, various calculation methods are proposed and developed. Nevertheless, most of the present calculation methods focus on specific geometries, e.g., plasmonic waveguide^26–28,32,33^, metallic nanoparticle^34–36^, spherical core–shell nanoparticle^37–39^, metallic resonator^40^, nanoparticle cluster^19,41^.

In this paper, we propose a general formalism to efficiently simulate the radiative coupling between two quantum emitters in arbitrary metallic nanostructures. More importantly, based on the radiative coupling, we propose an approach to calculate the evolution spectrum and population evolution of these two quantum emitters. As an illustrating application, we investigate the radiative coupling and decay dynamics of two quantum emitters embedded individually in the two nanogaps (hot spots) of three silver nano-spheroids. Due to the enormously enhanced electric field in the two hot spots, despite the strong plasmon damping, the strong radiative coupling between the two quantum emitters can still be manifested by the vacuum Rabi oscillation in population evolution and the anti-crossing behavior in evolution spectrum. This formalism may serve as a flexible and efficient theoretical tool for the distant coherent interaction between two quantum emitters in a large variety of metallic nanostructures, and may be further developed to handle the cases for multiple quantum emitters and arbitrary dielectric-metallic hybrid nanostructures.

## Results

### Theory

In this section, we deduce a formalism to calculate the temporal evolution of the upper-level-probability amplitudes of two two-level quantum emitters (denoted as $A$ and $B$) inside arbitrary metallic nanostructure. By adopting the dipole approximation and the rotating-wave approximation, the Hamiltonian of this system can be expressed as^37,42,43^

1 $$ \hat{H} = \int {d^{3} {\mathbf{r}}\int_{0}^{ + \infty } {d\omega \hbar \omega {\hat{\mathbf{f}}}^{\dag } ({\mathbf{r}},\omega ) \cdot {\hat{\mathbf{f}}}({\mathbf{r}},\omega )} } + \sum\limits_{i = A,B} {\hbar \omega_{i} \left| {e_{i} } \right\rangle \left\langle {e_{i} } \right|} - \sum\limits_{i = A,B} {[\hat{\sigma }_{i}^{\dag } {\hat{\mathbf{E}}}^{( + )} ({\mathbf{r}}_{i} ) \cdot {\mathbf{d}}_{i} + \hat{\sigma }_{i} {\hat{\mathbf{E}}}^{( - )} ({\mathbf{r}}_{i} ) \cdot {\mathbf{d}}_{i} ]} . $$

Here, the first term represents the field energy of the environment in the presence of the metallic nanostructure.${\hat{\mathbf{f}}}({\mathbf{r}},\omega )$ and ${\hat{\mathbf{f}}}^{\dag } ({\mathbf{r}},\omega )$ are the bosonic fields that represent the elementary (energy) excitations of the electromagnetic field both in the environment and the metallic nanostructure^44^.

The second term in the Hamiltonian represents the energy of the two quantum emitters. $\omega_{i}$ and ${\mathbf{d}}_{i} = d_{i} {\hat{\mathbf{d}}}_{i}$ are the transition frequency and transition dipole moment with magnitude $d_{i}$ and direction ${\hat{\mathbf{d}}}_{i}$, respectively, between the excited state $\left| {e_{i} } \right\rangle$ and the ground state $\left| {g_{i} } \right\rangle$ of the $ith$ ($i = A,B$) quantum emitter located at ${\mathbf{r}}_{i}$.

The third term in the Hamiltonian represents the interaction between the quantum emitters and the field excitations. $\hat{\sigma }_{i}^{\dag }$ and $\hat{\sigma }_{i}$ are Pauli operators of the ith quantum emitter. The electric field operator is separated into two parts as ${\hat{\mathbf{E}}}({\mathbf{r}}) = {\hat{\mathbf{E}}}^{( + )} ({\mathbf{r}}) + {\hat{\mathbf{E}}}^{( - )} ({\mathbf{r}})$, where ${\hat{\mathbf{E}}}^{( + )} ({\mathbf{r}}) = \int_{0}^{ + \infty } {d\omega {\hat{\mathbf{\underline {E}} }}({\mathbf{r}},\omega )}$ and ${\hat{\mathbf{E}}}^{( - )} ({\mathbf{r}}) = [{\hat{\mathbf{E}}}^{( + )} ({\mathbf{r}})]^{\dag }$.$\hat{{\mathbf{\underline {E}} }}({\mathbf{r}},\omega )$ is the electric field operator in the frequency domain^44^. The positive frequency part ${\hat{\mathbf{E}}}^{( + )} ({\mathbf{r}})$ can be expressed as^42^

2 $$ {\hat{\mathbf{E}}}^{( + )} \left( {\mathbf{r}} \right) = i\int_{0}^{ + \infty } {d\omega \sqrt {\frac{\hbar }{{\pi \varepsilon_{0} }}} \frac{{\omega^{2} }}{{c^{2} }}\int {d^{3} {\mathbf{r}}^{\prime}\sqrt {\varepsilon_{I} ({\mathbf{r}}^{\prime},\omega )} {\mathbf{G}}({\mathbf{r}},{\mathbf{r}}^{\prime},\omega ) \cdot {\hat{\mathbf{f}}}({\mathbf{r}}^{\prime},\omega )} } . $$

Here, $\varepsilon_{I} ({\mathbf{r}},\omega )$ is the imaginary part of the complex relative permittivity $\varepsilon ({\mathbf{r}},\omega )$ of the metallic nanostructure.${\mathbf{G}}({\mathbf{r}},{\mathbf{r}}^{\prime},\omega )$ is the classical Green function (tensor), describing the system response at ${\mathbf{r}}$ to a point source at ${\mathbf{r}}^{\prime}$, and satisfying the equation

3 $$ \left[\frac{{\omega^{2} }}{{c^{2} }}\varepsilon ({\mathbf{r}},\omega ) - \nabla \times \nabla \times \right]{\mathbf{G}}({\mathbf{r}},{\mathbf{r}}^{\prime},\omega ) = - {{\varvec{\updelta}}}({\mathbf{r}} - {\mathbf{r}}^{\prime}) $$

together with the boundary condition at infinity. ${{\varvec{\updelta}}}({\mathbf{r}})$ is the dyadic $\delta$ function.

We denote the system states as $\left| a \right\rangle = \left| {e_{A} ,g_{B} ,0} \right\rangle$, $\left| b \right\rangle = \left| {g_{A} ,e_{B} ,0} \right\rangle$ and $\left| {m({\mathbf{r}},\omega )} \right\rangle = \left| {g_{A} ,g_{B} ,1_{m} ({\mathbf{r}},\omega )} \right\rangle$, with only one excitation at quantum emitter $A$, $B$ or the bosonic field $\hat{f}_{m} ({\mathbf{r}},\omega )$ ($m$ denote the $x$, $y$ or $z$ component), respectively. The system state evolves as

4 $$ \left| {\Psi (t)} \right\rangle = C_{a} (t)\left| a \right\rangle + C_{b} (t)\left| b \right\rangle + \int {d^{3} {\mathbf{r}}\int_{0}^{ + \infty } {d\omega \sum\limits_{m = x,y,z} {C_{m} ({\mathbf{r}},\omega ,t)\left| {m({\mathbf{r}},\omega )} \right\rangle } } } . $$

Here, $C_{a} (t)$, $C_{b} (t)$ and $C_{m} ({\mathbf{r}},\omega ,t)$ are the probability amplitudes of $\left| a \right\rangle$, $\left| b \right\rangle$ and $\left| {m({\mathbf{r}},\omega )} \right\rangle$, respectively.

By substituting Eqs. (1), (2) and (4) into Schrödinger equation $i\hbar \frac{\partial }{\partial t}\left| {\Psi (t)} \right\rangle = \hat{H}\left| {\Psi (t)} \right\rangle$, and considering the orthonormal relationship among $\left| a \right\rangle$, $\left| b \right\rangle$ and $\left| {m({\mathbf{r}},\omega )} \right\rangle$, we can obtain

5 $$ \dot{C}_{a} (t) = - i\omega_{A} C_{a} (t) - \int {d^{3} {\mathbf{r}}\int_{0}^{ + \infty } {d\omega \sqrt {\frac{1}{{\hbar \pi \varepsilon_{0} }}} \frac{{\omega^{2} }}{{c^{2} }}\sqrt {\varepsilon_{I} ({\mathbf{r}},\omega )} \sum\limits_{m,n} {d_{An} G_{nm} ({\mathbf{r}}_{A} ,{\mathbf{r}},\omega )C_{m} ({\mathbf{r}},\omega ,t)} } } , $$

6 $$ \dot{C}_{b} (t) = - i\omega_{B} C_{b} (t) - \int {d^{3} {\mathbf{r}}\int_{0}^{ + \infty } {d\omega \sqrt {\frac{1}{{\hbar \pi \varepsilon_{0} }}} \frac{{\omega^{2} }}{{c^{2} }}\sqrt {\varepsilon_{I} ({\mathbf{r}},\omega )} \sum\limits_{m,n} {d_{Bn} G_{nm} ({\mathbf{r}}_{B} ,{\mathbf{r}},\omega )C_{m} ({\mathbf{r}},\omega ,t)} } } , $$

7 $$ \dot{C}_{m} ({\mathbf{r}},\omega ,t) = - i\omega C_{m} ({\mathbf{r}},\omega ,t) + \sqrt {\frac{1}{{\hbar \pi \varepsilon_{0} }}} \frac{{\omega^{2} }}{{c^{2} }}\sqrt {\varepsilon_{I} ({\mathbf{r}},\omega )} \sum\limits_{n} {[d_{An} G_{nm}^{*} ({\mathbf{r}}_{A} ,{\mathbf{r}},\omega )C_{a} (t) + d_{Bn} G_{nm}^{*} ({\mathbf{r}}_{B} ,{\mathbf{r}},\omega )C_{b} (t)]} . $$

We assume that initially there is only one excitation at the two quantum emitters and no excitation at the bosonic fields, i.e., the probability amplitudes for the initial state of the system are

8 $$ \begin{gathered} \left| {C_{a} (0)} \right|^{2} + \left| {C_{b} (0)} \right|^{2} = 1, \hfill \\ C_{m} ({\mathbf{r}},\omega ,0) = 0. \hfill \\ \end{gathered} $$

We take the Laplace transform (forward and backward Fourier transform) to transform Eqs. (5)–(7) into algebraic equations. The forward and backward Fourier transform of any time-dependent variable $C(t)$, e.g., $C_{a} (t)$, $C_{b} (t)$ and $C_{m} ({\mathbf{r}},\omega ,t)$, can be defined, respectively, as

9 $$ c^{ + } (\Omega^{ + } ) = \int_{0}^{ + \infty } {C(t)e^{{i\Omega^{ + } t}} dt} , $$

10 $$ c^{ - } (\Omega^{ - } ) = \int_{ - \infty }^{0} {C(t)e^{{i\Omega^{ - } t}} dt} . $$

Here, the complex frequencies $\Omega^{ \pm } = \Omega \pm i\eta$ are assumed to contain a real frequency $\Omega$ and an infinitely small positive and negative imaginary part $\pm i\eta$($\eta \to 0^{ + }$), respectively, so that the transform is well-defined^45^.

As derived in the “Methods” section, the forward and backward Fourier transform of $C_{a} (t)$ and $C_{b} (t)$ can be calculated as

11 $$ c_{a}^{ \pm } (\Omega^{ \pm } ) = \pm i\frac{{[\Omega^{ \pm } - \omega_{B} - W_{BB}^{ \pm } (\Omega )]C_{a} (0) + W_{AB}^{ \pm } (\Omega )C_{b} (0)}}{{[\Omega^{ \pm } - \omega_{A} - W_{AA}^{ \pm } (\Omega )][\Omega^{ \pm } - \omega_{B} - W_{BB}^{ \pm } (\Omega )] - W_{AB}^{ \pm } (\Omega )W_{BA}^{ \pm } (\Omega )}}, $$

12 $$ c_{b}^{ \pm } (\Omega^{ \pm } ) = \pm i\frac{{[\Omega^{ \pm } - \omega_{A} - W_{AA}^{ \pm } (\Omega )]C_{b} (0) + W_{BA}^{ \pm } (\Omega )C_{a} (0)}}{{[\Omega^{ \pm } - \omega_{A} - W_{AA}^{ \pm } (\Omega )][\Omega^{ \pm } - \omega_{B} - W_{BB}^{ \pm } (\Omega )] - W_{AB}^{ \pm } (\Omega )W_{BA}^{ \pm } (\Omega )}}. $$

Here, as derived in the “Methods” section and discussed in detail later, the coupling matrix element is

13 $$ W_{ij}^{ \pm } (\Omega ) = \Delta_{ij} (\Omega ) \mp i\frac{{\Gamma_{ij} (\Omega )}}{2} = - \frac{{\Omega^{2} }}{{\varepsilon_{0} \hbar c^{2} }}{\mathbf{d}}_{i} \cdot {\mathbf{G}}^{/*} ({\mathbf{r}}_{i} ,{\mathbf{r}}_{j} ,\Omega ) \cdot {\mathbf{d}}_{j} . $$

Here, ${\mathbf{G}}^{/*}$ denote ${\mathbf{G}}$ and ${\mathbf{G}}^{*}$, corresponding to $W_{ij}^{ + }$ and $W_{ij}^{ - }$, respectively.

Furthermore, the evolution spectrum of the two quantum emitters can be obtained as

14 $$ c_{a} (\Omega ) = c_{a}^{ + } (\Omega^{ + } ) + c_{a}^{ - } (\Omega^{ - } ), $$

15 $$ c_{b} (\Omega ) = c_{b}^{ + } (\Omega^{ + } ) + c_{b}^{ - } (\Omega^{ - } ). $$

Finally, the temporal evolution of the two quantum emitters can be calculated via their evolution spectrum as

16 $$ C_{a} (t) = \frac{1}{2\pi }\int_{ - \infty }^{ + \infty } {c_{a} (\Omega )e^{ - i\Omega t} d\Omega } , $$

17 $$ C_{b} (t) = \frac{1}{2\pi }\int_{ - \infty }^{ + \infty } {c_{b} (\Omega )e^{ - i\Omega t} d\Omega } . $$

Obviously, the key to investigate the decay dynamics of two quantum emitters in arbitrary metallic nanostructure is the calculation of the coupling matrix element $W_{ij}^{ \pm } (\Omega )$, which can be obtained via Green tensor ${\mathbf{G}}({\mathbf{r}}_{i} ,{\mathbf{r}}_{j} ,\Omega )$ according to Eq. (13). ${\mathbf{G}}({\mathbf{r}}_{i} ,{\mathbf{r}}_{j} ,\Omega )$ describes the system response at ${\mathbf{r}}_{i}$ (the *i*th quantum emitter) to a point source at ${\mathbf{r}}_{j}$ (the *j*th quantum emitter) and characterizes the electromagnetic environment of the two quantum emitters, which determines the radiative coupling between them.

For specific metallic nanostructures with simple geometries, e.g., spherical and spheroidal geometries^46^, ${\mathbf{G}}({\mathbf{r}}_{i} ,{\mathbf{r}}_{j} ,\Omega )$ can be obtained analytically. However, for arbitrary metallic nanostructures with arbitrary geometries and components, numerical calculations should be adopted to obtain ${\mathbf{G}}({\mathbf{r}}_{i} ,{\mathbf{r}}_{j} ,\Omega )$. Actually, the Green tensor ${\mathbf{G}}({\mathbf{r}}_{i} ,{\mathbf{r}}_{j} ,\Omega )$ and hence the coupling matrix element $W_{ij}^{ \pm } (\Omega )$ can be obtained by simulating the electric fields induced by two point-dipoles, individually.

From Maxwell equations, the electric field induced by an oscillating point-dipole (related to the *j*th quantum emitter) located at ${\mathbf{r}}_{j}$ with unit magnitude, direction ${\hat{\mathbf{d}}}_{j}$ and frequency $\Omega$ is^47,48^

18 $$ {\mathbf{E}}_{j} ({\mathbf{r}},\Omega ) = \frac{{\Omega^{2} }}{{\varepsilon_{0} c^{2} }}{\mathbf{G}}({\mathbf{r}},{\mathbf{r}}_{j} ,\Omega ) \cdot {\hat{\mathbf{d}}}_{j} . $$

Specially, the electric field component at location ${\mathbf{r}}_{i}$ and along direction ${\hat{\mathbf{d}}}_{i}$ (related to the $ith$ quantum emitter) is

19 $$ {\hat{\mathbf{d}}}_{i} \cdot {\mathbf{E}}_{j} ({\mathbf{r}}_{i} ,\Omega ) = \frac{{\Omega^{2} }}{{\varepsilon_{0} c^{2} }}{\hat{\mathbf{d}}}_{i} \cdot {\mathbf{G}}({\mathbf{r}}_{i} ,{\mathbf{r}}_{j} ,\Omega ) \cdot {\hat{\mathbf{d}}}_{j} . $$

According to the definition of Eq. (13), we can rewrite the coupling matrix element as

20 $$ W_{ij}^{ \pm } (\Omega ) = - \frac{{d_{i} d_{j} }}{\hbar }{\hat{\mathbf{d}}}_{i} \cdot {\mathbf{E}}_{j}^{/*} ({\mathbf{r}}_{i} ,\Omega ). $$

Here, ${\mathbf{E}}_{j}^{/*}$ denote ${\mathbf{E}}_{j}$ and ${\mathbf{E}}_{j}^{*}$, corresponding to $W_{ij}^{ + }$ and $W_{ij}^{ - }$, respectively. This relationship provides a flexible and efficient approach to calculate the coupling matrix element $W_{ij}^{ \pm } (\Omega )$, based on the numerical simulation of the electric fields ${\mathbf{E}}_{j} ({\mathbf{r}}_{i} ,\Omega )$ induced by two individual point-dipoles.

${\mathbf{E}}_{j} ({\mathbf{r}}_{i} ,\Omega )$ in arbitrary metallic nanostructure can be simulated directly in frequency domain, e.g., by the COMSOL Multiphysics. Alternatively, at first, ${\mathbf{E}}_{j} ({\mathbf{r}}_{i} ,t)$ in time domain can be simulated, e.g., via the finite-difference time-domain (FDTD) method^49^, and then ${\mathbf{E}}_{j} ({\mathbf{r}}_{i} ,\Omega )$ can be obtained by Fourier transform or Padé approximation with Baker’s algorithm^50^.

For $i \ne j$, i.e., $W_{AB}^{ \pm } (\Omega )$ and $W_{BA}^{ \pm } (\Omega )$, $W_{ij}^{ \pm } (\Omega ) = \Delta_{ij} (\Omega ) \mp i\frac{{\Gamma_{ij} (\Omega )}}{2}$ characterizes the radiative coupling between the *i*th and the *j*th quantum emitters, mediated by the electromagnetic field (mainly the plasmon) in the metallic nanostructures. The real and imaginary part of $W_{ij}^{ \pm } (\Omega )$ corresponds to the collective level shift $\Delta_{ij} (\Omega )$ and the transfer rate $\Gamma_{ij} (\Omega )$, respectively^26,32,51^. $W_{ij}^{ \pm } (\Omega )$ can be directly calculated from the complex ${\mathbf{E}}_{j} ({\mathbf{r}}_{i} ,\Omega )$ via Eq. (20). Obviously, $W_{ij}^{ - } (\Omega )$ is the complex conjugate of $W_{ij}^{ + } (\Omega )$. Besides, according to Eqs. (13) and (20), we can separately calculate $\Delta_{ij} (\Omega )$ and $\Gamma_{ij} (\Omega )$ via the real and imaginary part of ${\mathbf{E}}_{j} ({\mathbf{r}}_{i} ,\Omega )$, respectively, as

21 $$ \Delta_{ij} (\Omega ) = - \frac{{d_{i} d_{j} }}{\hbar }{\hat{\mathbf{d}}}_{i} \cdot {\text{Re}} [{\mathbf{E}}_{j} ({\mathbf{r}}_{i} ,\Omega )], $$

22 $$ \Gamma_{ij} (\Omega ) = \frac{{2d_{i} d_{j} }}{\hbar }{\hat{\mathbf{d}}}_{i} \cdot {\text{Im}} [{\mathbf{E}}_{j} ({\mathbf{r}}_{i} ,\Omega )]. $$

For $i = j$, i.e., $W_{AA}^{ \pm } (\Omega )$ and $W_{BB}^{ \pm } (\Omega )$, $W_{ii}^{ \pm } (\Omega ) = \Delta_{ii} (\Omega ) \mp i\frac{{\Gamma_{ii} (\Omega )}}{2}$ characterizes the local coupling between the *i*th quantum emitter and the electromagnetic field (mainly the plasmon) in the metallic nanostructure. The real and imaginary part of $W_{ii}^{ \pm } (\Omega )$ corresponds to the level shift $\Delta_{ii} (\Omega )$ and the local coupling strength $\Gamma_{ii} (\Omega )$ of the *i*th quantum emitter, respectively. Unfortunately, $W_{ii}^{ \pm } (\Omega )$ can’t be directly calculated via Eq. (20). Instead, at first, its imaginary part can be calculated via $\Gamma_{ii} (\Omega ) = \frac{{2d_{i}^{2} }}{\hbar }{\hat{\mathbf{d}}}_{i} \cdot {\text{Im}} [{\mathbf{E}}_{i} ({\mathbf{r}}_{i} ,\Omega )]$, then its real part can be calculated via the principal value integral as $\Delta_{ii} (\Omega ) = \frac{1}{2\pi }\mathcal{P}\int_{0}^{{{ + }\infty }} {d\omega \frac{{\Gamma_{ii} (\omega )}}{\Omega - \omega }}$^52,53^. Besides, $W_{ii}^{ - } (\Omega )$ is the complex conjugate of $W_{ii}^{ + } (\Omega )$.

With the calculated $W_{AB}^{ \pm } (\Omega )$, $W_{BA}^{ \pm } (\Omega )$, $W_{AA}^{ \pm } (\Omega )$ and $W_{BB}^{ \pm } (\Omega )$, via Eqs. (11) and (12), we can successively obtain the evolution spectrum of (14) and (15) and the temporal evolution of Eqs. (16) and (17).

In plasmonic systems, the dominant mechanism is surface plasmon exchange, i.e., excitation of a virtual surface plasmon in the metallic nanostructure by an excited quantum emitter followed by its absorption by the other quantum emitter, rather than direct radiative coupling^31^. The simulated electric field ${\mathbf{E}}_{j} ({\mathbf{r}}_{i} ,\Omega )$ corresponds to the excitation and absorption of a virtual surface plasmon. Actually, all system responses including the dominating surface plasmon and other minor electromagnetic responses are totally incorporated in the simulated electric field ${\mathbf{E}}_{j} ({\mathbf{r}}_{i} ,\Omega )$. So this formalism is accurate in calculating the radiative coupling between two quantum emitters.

This formalism is flexible and efficient. It can simulate the radiative coupling and decay dynamics of two quantum emitters at any location, along any polarization direction, with any transition frequency, inside arbitrary metallic nanostructure including, but not limited to, plasmonic cavity and plasmonic waveguide. Besides, only the electric fields at the locations of the two quantum emitters need to be simulated, rather than those of the whole space. This saves massive computational time and memory.

### Simulation

**Figure 1:**
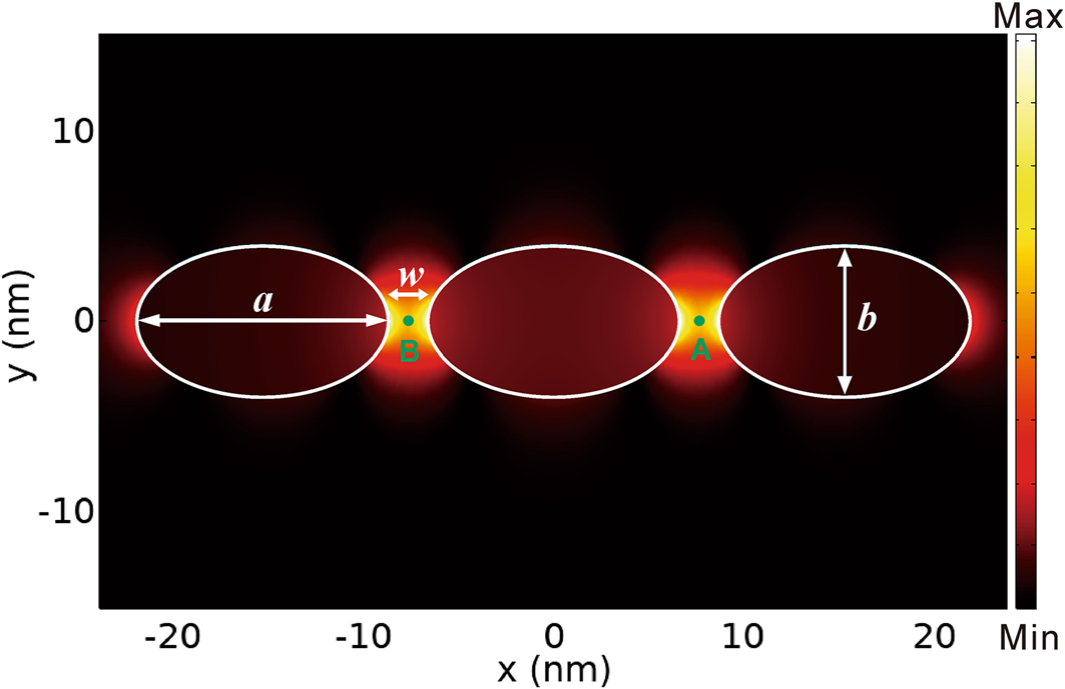
The three nano-spheroid structure and its cavity mode ($x$ component of electric field on $z = 0$ plane) with resonant wavelength $\lambda_{r} = 525.6\,{\text{nm}}$. The three white ellipses denote the three silver nano-spheroids. The two green dots denote the locations of quantum emitter $A$ and $B$.

To demonstrate and verify this formalism, we apply it to investigate the radiative coupling and decay dynamics of two quantum emitters in the metallic nanostructure composed of three silver nano-spheroids optimized in Ref.^20^. As shown in Fig. 1, three identical silver nano-spheroids are lined along their elongated axis (along $x$ direction) with axis length $a = 13.3\,{\text{nm}}$. The other two orthogonal axes (along $y$ and $z$ directions) have the same axis length $b = 8\,{\text{nm}}$. The gap widths between any two-neighboring nano-spheroids along the $x$ direction is $w = 2\,{\text{nm}}$. The center of the middle nano-spheroid locates at the origin of the coordinate system. The three nano-spheroids are embedded inside lossless host medium of dielectric constant $\varepsilon_{h} = 2.2$. Here the refractive index parameters of Ag are obtained by interpolation from the original experimental data in the literature^54^. At the resonant wavelength $\lambda_{r} = 525.6\,{\text{nm}}$ (resonant frequency $\hbar \Omega_{r} = 2.3589\,{\text{eV}}$) of this metallic nanostructure, the electric field are strongly confined and enhanced inside the two gaps, forming two hot spots of electric field, as shown in Fig. 1.

To achieve strong radiative coupling, according to Eq. (20), the two quantum emitters should be individually positioned at the hot spots of electric field. In this three nano-spheroid structure, a natural choice for the locations of the two quantum emitters is the two gap centers, $w/2$ away from the neighboring nano-spheroids. we take the transition dipole moment of the two quantum emitters as $d_{A} = d_{B} = {4}{\text{.167}} \times 10^{ - 29} \,{\text{C}}\,{\text{m}}$, corresponding to the lifetimes in the homogeneous host medium is $\tau_{A} = \tau_{B} = 2\,{\text{ns}}$. The transition dipole moment of the two quantum emitters are both polarized along $x$ direction to obtain the strongest radiative coupling.

**Figure 2:**
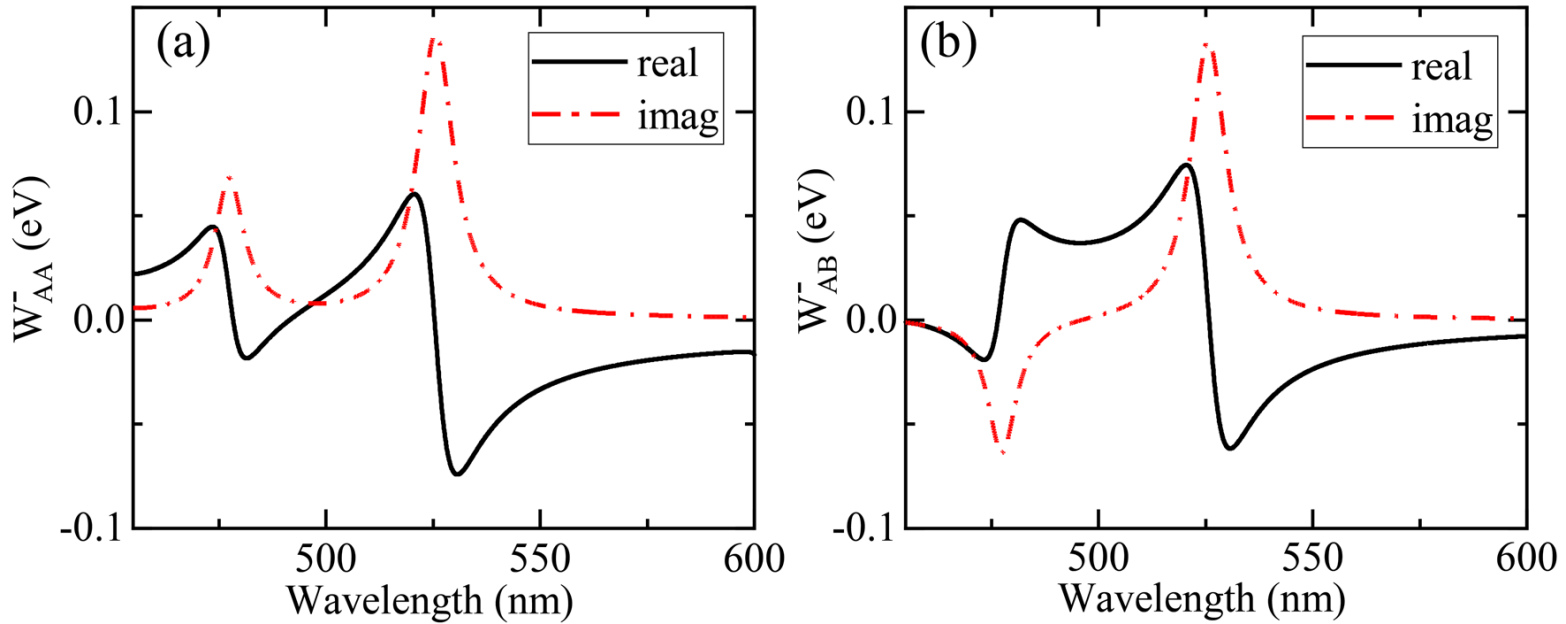
The real and imaginary parts of the coupling matrix element (**a**) $W_{AA}^{ - } (\Omega ) = \Delta_{AA} (\Omega ) + i\frac{{\Gamma_{AA} (\Omega )}}{2}$ and (**b**) $W_{AB}^{ - } (\Omega ) = \Delta_{AB} (\Omega ) + i\frac{{\Gamma_{AB} (\Omega )}}{2}$ for different frequency $\Omega$ (wavelength $\lambda$).

Based on the electric fields induced by two individual dipole (with unit magnitude, at the locations and along the polarizations of the two quantum emitters, with different frequency), via Eq. (20) and principal value integral, we calculate the coupling matrix element $W_{ij}^{ \pm } (\Omega )$ between quantum emitter $A$ and $B$, as shown in Fig. 2. For any $i$ and $j$, $W_{ij}^{ - } (\Omega )$ is the complex conjugate of $W_{ij}^{ + } (\Omega )$. Furthermore, since the three nano-spheroid structure is symmetrical and the two quantum emitters have the same transition dipole moments, we can obtain $W_{AA}^{ \pm } (\Omega ) = W_{BB}^{ \pm } (\Omega )$ and $W_{AB}^{ \pm } (\Omega ) = W_{BA}^{ \pm } (\Omega )$.

For the imaginary parts related to the local coupling strength $\Gamma_{AA} (\Omega )$ in Fig. 2a and to the transfer rate $\Gamma_{AB} (\Omega )$ in Fig. 2b, there are both two resonant peaks (dips) at $525.6\,{\text{nm}}$ and $477.45\,{\text{nm}}$. For resonant wavelength of $525.6\,{\text{nm}}$ and $477.45\,{\text{nm}}$, the electric field distribution is symmetric and antisymmetric about the $x = 0$ plane, respectively. We focus on the symmetric mode of resonant wavelength $\lambda_{r} = 525.6\,{\text{nm}}$, referred to as cavity mode, as shown in Fig. 1. The linewidth for its resonant peak is $10.22\,{\text{nm}}$ (cavity leakage $\hbar \kappa = 45.9\,{\text{meV}}$).

In this paper, we focus on the ideal case that the two quantum emitters have the same transition wavelength $\lambda_{A} = \lambda_{B}$. Besides, we assume that at initial time $t = 0$, quantum emitter $A$ is in excited state, quantum emitter $B$ is in ground state, and there is no excitation at the bosonic fields, i.e., the initial state of the system is $C_{a} (0) = 1$, $C_{b} (0) = 0$, $C_{m} ({\mathbf{r}},\omega ,0) = 0$.

**Figure 3:**
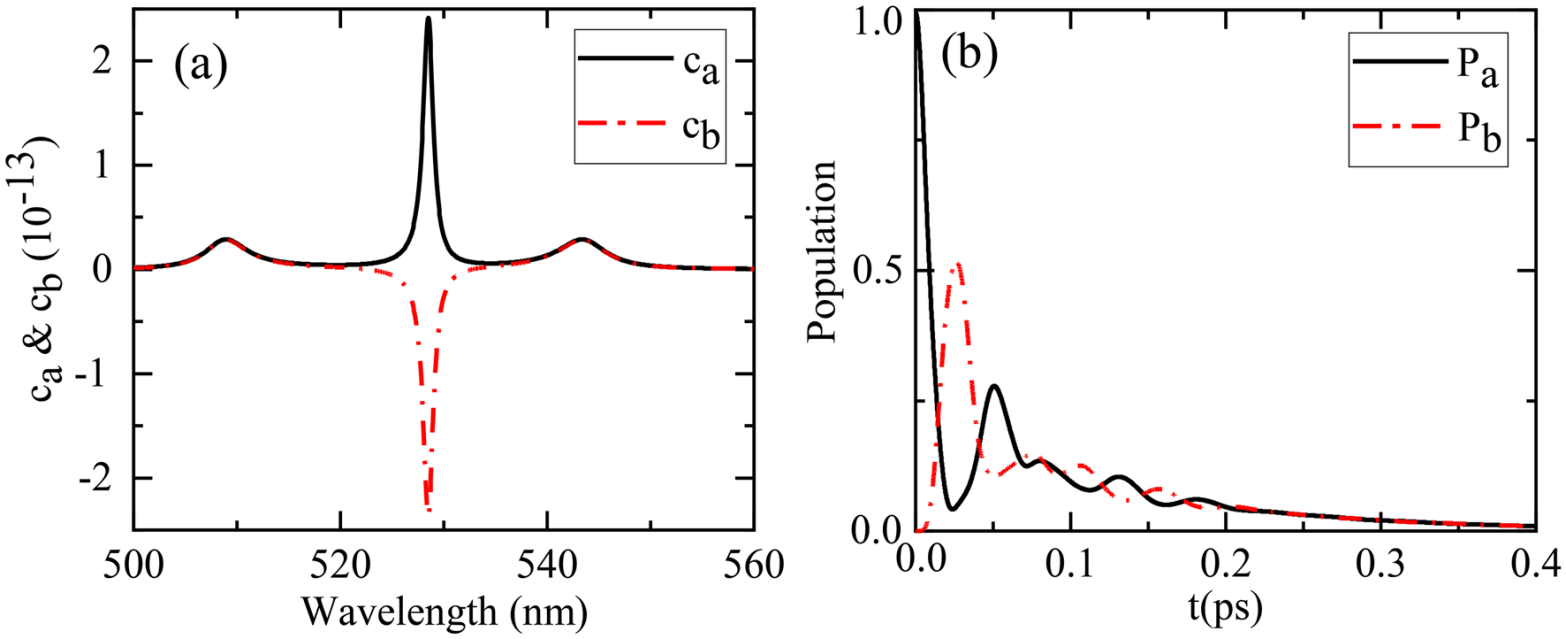
Decay dynamics of the two quantum emitters for resonance case $\lambda_{A} = \lambda_{B} = \lambda_{r} = 525.6\,{\text{nm}}$. (**a**) Evolution spectrum $c_{a} (\Omega )$ and $c_{b} (\Omega )$. (**b**) Population $P_{a} (t)$ and $P_{b} (t)$.

At first, we consider the resonance case that the two quantum emitters are both resonant with the three nano-spheroid structure, i.e., $\lambda_{A} = \lambda_{B} = \lambda_{r} = 525.6\,{\text{nm}}$. By adopting Eqs. (11), (12), (14) and (15), we calculate the evolution spectrum of the two quantum emitters, as shown in Fig. 3a. Since $C_{a} (0)$ and $C_{b} (0)$ are both real, we conclude from Eqs. (11) and (12) that $c_{a}^{ + } (\Omega^{ + } )$ and $c_{b}^{ + } (\Omega^{ + } )$ are complex conjugate of $c_{a}^{ - } (\Omega^{ - } )$ and $c_{b}^{ - } (\Omega^{ - } )$, respectively. In this case, according to Eqs. (14) and (15), the evolution spectrum of the two quantum emitters $c_{a} (\Omega )$ and $c_{b} (\Omega )$ are both real.

As shown in Fig. 3a, there are three peaks (dips) in the evolution spectrum $c_{a} (\Omega )$ and $c_{b} (\Omega )$, respectively. The central peak (dip) at wavelength $528.5\,{\text{nm}}$ (near $\lambda_{A} = \lambda_{B} = 525.6\,{\text{nm}}$) is the dark mode, i.e., the antisymmetric state of the two quantum emitters. The central peak is relatively narrow since the linewidths of the two quantum emitters are negligible. In contrast, the two side peaks (corresponding to the upper and lower polaritons) at $508.95\,{\text{nm}}$ and $543.35\,{\text{nm}}$, with splitting of $34.4\,{\text{nm}}$ ($154\,{\text{meV}}$), are the bright modes composed of the symmetric state of the two quantum emitters and the cavity mode. The linewidths of the two side peaks are relatively large due to the large linewidth of the cavity mode. The large splitting between the two side peaks results from the strong radiative coupling between the two quantum emitters, mediated by the plasmon of this three nano-spheroids structure with extremely strong electric field in the hot spots.

The strong radiative coupling is most apparent in time domain. Based on the evolution spectrum $c_{a} (\Omega )$ and $c_{b} (\Omega )$, via Eqs. (16) and (17), we further obtain the time-dependent probabilities of excitation, i.e., the populations of quantum emitter $A$ and $B$ as $P_{a} (t) = \left| {C_{a} (t)} \right|^{2}$ and $P_{b} (t) = \left| {C_{b} (t)} \right|^{2}$. As shown in Fig. 3b, initially, quantum emitter $A$ is in excited state and quantum emitter $B$ is in ground state. For $t > 0$, due to the strong radiative coupling between the two quantum emitters mediated by the plasmon (cavity mode), the populations of the two quantum emitters both oscillate at the frequency of $\hbar g = 80.2\,\,{\text{meV}}$, about half of the splitting ($154\,\,{\text{meV}}$) between the two side peaks in Fig. 3a. The excitation energy is coherently transferred between the two quantum emitters. Meanwhile, due to the cavity leakage $\hbar \kappa = 45.9\,\,{\text{meV}}$, the amplitudes of the two oscillating populations damp quickly to zero in picosecond scale. The excitation energy finally dissipates mainly due to the ohmic loss of the metallic nanostructure. This pronounced population oscillation is a clear signature of strong radiative coupling, which occurs in a regime where the radiative coupling $g$ is stronger than the plasmon damping $\kappa$.

Now we turn to investigate the off-resonance case by simultaneously tune the transition wavelength of the two quantum emitters (keeping $\lambda_{A} = \lambda_{B}$ and $\lambda_{r} = 525.6\,{\text{nm}}$), and calculate the evolution spectrum $c_{a} (\Omega )$ and $c_{b} (\Omega )$ for different emitter-cavity detuning $\lambda_{A} - \lambda_{r}$.

**Figure 4:**
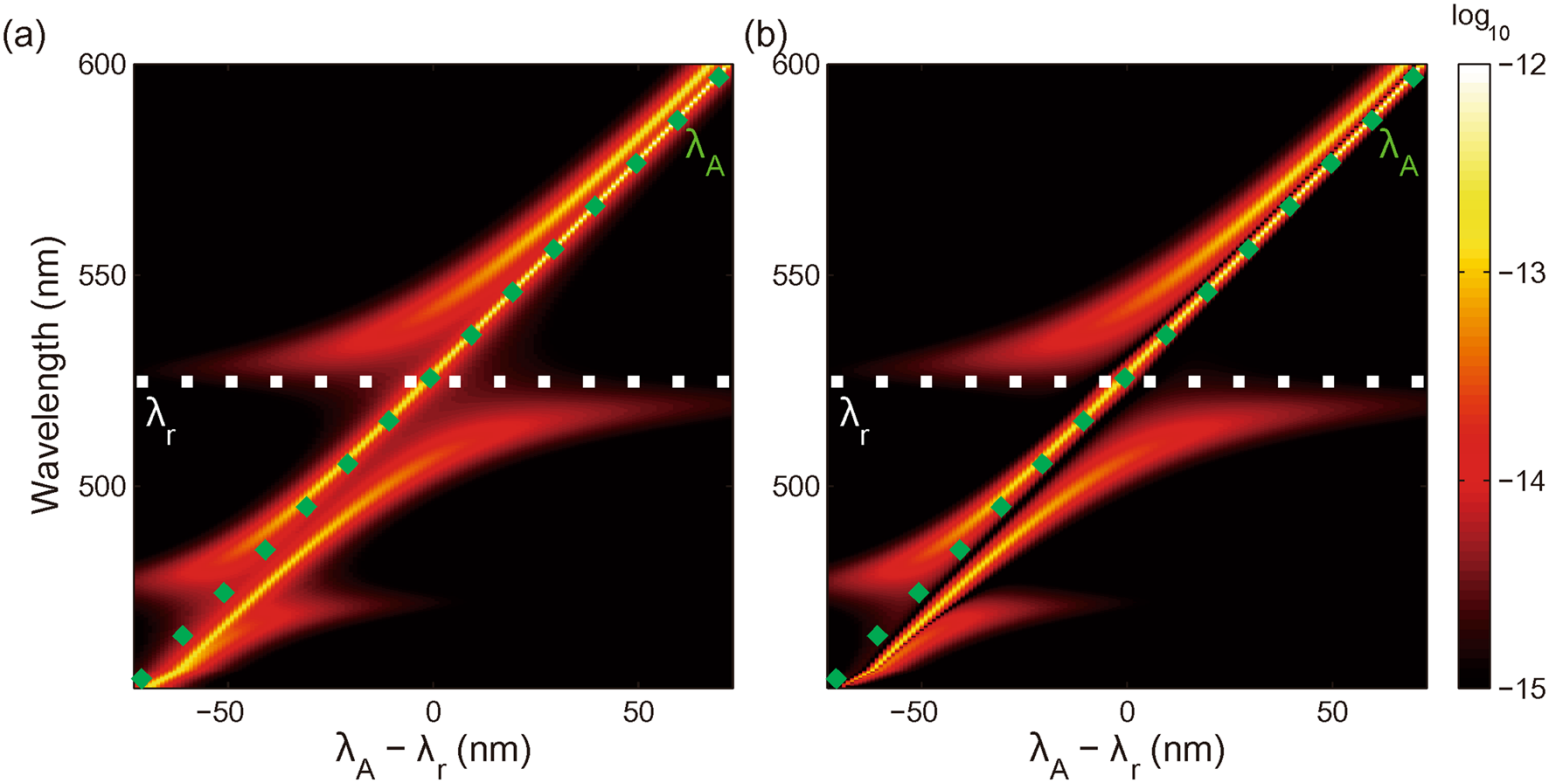
Evolution spectrum (**a**) $c_{a} (\Omega )$ and (**b**) $\left| {c_{b} (\Omega )} \right|$ for off-resonance case $\lambda_{A} = \lambda_{B} \ne \lambda_{r}$. The dotted white line denotes constant $\lambda_{r} = 525.6\,{\text{nm}}$. The dotted green line denotes varying $\lambda_{A}$($= \lambda_{B}$).

As shown in Fig. 4, for positively large emitter-cavity detuning $\lambda_{A} - \lambda_{r}$, there are three peaks in both $c_{a} (\Omega )$ and $c_{b} (\Omega )$. The central peak at $\lambda_{A}$ is dark mode, i.e., the antisymmetric state of the two quantum emitters. The side peak approaching $\lambda_{A}$ can be mainly attributed to the quantum emitter. The other side peak approaching $\lambda_{r}$ can be mainly attributed to the cavity mode. The attribution of the three peaks can also be verified by their linewidths comparing with those of the quantum emitters and cavity mode.

As emitter-cavity detuning $\lambda_{A} - \lambda_{r}$ decreases, the two side peaks gradually repel each other and can both be attributed to the two quantum emitters and the cavity mode. This behavior is quite similar to the strong coupling system composed of a single quantum emitter and metallic nanostructure^55^.

For zero emitter-cavity detuning, i.e., $\lambda_{A} = \lambda_{B} = \lambda_{r} = 525.6\,{\text{nm}}$, the two side peaks forms two polaritonic states, which is the case of Fig. 3a.

For negatively large emitter-cavity detuning $\lambda_{A} - \lambda_{r}$, the evolution spectrum $c_{a} (\Omega )$ and $c_{b} (\Omega )$ are both interfered by the other resonant peak at $477.45\,{\text{nm}}$, i.e., the antisymmetric mode in Fig. 2. It leads to an extra minor anti-crossing behavior.

## Discussion

In this paper, we have proposed a general formalism to calculate the plasmon-mediated radiative coupling between two quantum emitters in arbitrary metallic nanostructure. The coupling matrix element $W_{ij}^{ \pm } (\Omega )$ can be flexibly and efficiently calculated by simulating the electric fields ${\mathbf{E}}_{j} ({\mathbf{r}}_{i} ,\Omega )$ induced by two point-dipoles, individually. Based on the coupling matrix element $W_{ij}^{ \pm } (\Omega )$, the evolution spectrum and population evolution of the two quantum emitters can be obtained.

We have demonstrated this formalism to investigate the radiative coupling and decay dynamics of two quantum emitters located in the two hot spots of three silver nano-spheroids. The vacuum Rabi oscillation in population evolution and the anti-crossing behavior in evolution spectrum are clearly observed for both quantum emitters. Obviously, despite the strong plasmon damping, the strong radiative coupling between the two quantum emitters can still be realized in this metallic nanostructure, due to the enormously enhanced electric field in the two hot spots.

This formalism can serve as a flexible and efficient calculation tool to investigate the distant coherent interaction between two quantum emitters in a large variety of metallic nanostructures including, but not limited to, plasmonic cavities and plasmonic waveguides. Besides, it can be further developed to simulate the cases for multiple quantum emitters, which is essential for multiqubit manipulation. It can also be further developed to simulate the case for other nanostructures with arbitrary geometries and components, e.g., the hybrid nanostructures composed of both dielectric and metallic components^56^, where the strong radiative coupling and long-time coherence might be simultaneously realized for quantum information processing.

## Methods

### Derivation of Eqs. (11) and (12)

According to the definition of Eq. (9), the forward Fourier transform of $\dot{C}(t)$ is

23 $$ \int_{0}^{ + \infty } {\dot{C}(t){\text{e}}^{{i\Omega^{ + } t}} {\text{d}}t} = - C(0) - i\Omega^{ + } c^{ + } (\Omega^{ + } ). $$

Performing forward Fourier transform to Eqs. (5)–(7), and regarding the initial condition of Eq. (8), we can obtain

24 $$ - C_{a} (0) - i(\Omega^{ + } - \omega_{A} )c_{a}^{ + } (\Omega^{ + } ) = - \int {d^{3} {\mathbf{r}}\int_{0}^{ + \infty } {d\omega \sqrt {\frac{1}{{\hbar \pi \varepsilon_{0} }}} \frac{{\omega^{2} }}{{c^{2} }}\sqrt {\varepsilon_{I} ({\mathbf{r}},\omega )} \sum\limits_{m,n} {d_{An} G_{nm} ({\mathbf{r}}_{A} ,{\mathbf{r}},\omega )c_{m}^{ + } ({\mathbf{r}},\omega ,\Omega^{ + } )} } } , $$

25 $$ - C_{b} (0) - i(\Omega^{ + } - \omega_{B} )c_{b}^{ + } (\Omega^{ + } ) = - \int {d^{3} {\mathbf{r}}\int_{0}^{ + \infty } {d\omega \sqrt {\frac{1}{{\hbar \pi \varepsilon_{0} }}} \frac{{\omega^{2} }}{{c^{2} }}\sqrt {\varepsilon_{I} ({\mathbf{r}},\omega )} \sum\limits_{m,n} {d_{Bn} G_{nm} ({\mathbf{r}}_{B} ,{\mathbf{r}},\omega )c_{m}^{ + } ({\mathbf{r}},\omega ,\Omega^{ + } )} } } , $$

26 $$ c_{m}^{ + } ({\mathbf{r}},\omega ,\Omega^{ + } ) = \frac{{\sqrt {\frac{1}{{\hbar \pi \varepsilon_{0} }}} \frac{{\omega^{2} }}{{c^{2} }}\sqrt {\varepsilon_{I} ({\mathbf{r}},\omega )} \sum\limits_{n} {[d_{An} G_{nm}^{*} ({\mathbf{r}}_{A} ,{\mathbf{r}},\omega )c_{a}^{ + } (\Omega^{ + } ) + d_{Bn} G_{nm}^{*} ({\mathbf{r}}_{B} ,{\mathbf{r}},\omega )c_{b}^{ + } (\Omega^{ + } )]} }}{{ - i(\Omega^{ + } - \omega )}}. $$

Substituting Eq. (26) into Eq. (24) and Eq. (25), respectively, we can obtain

27 $$ c_{a}^{ + } (\Omega^{ + } ) = \frac{{iC_{a} (0) + W_{AB}^{ + } (\Omega )c_{b}^{ + } (\Omega^{ + } )}}{{\Omega^{ + } - \omega_{A} - W_{AA}^{ + } (\Omega )}}, $$

28 $$ c_{b}^{ + } (\Omega^{ + } ) = \frac{{iC_{b} (0) + W_{BA}^{ + } (\Omega )c_{a}^{ + } (\Omega^{ + } )}}{{\Omega^{ + } - \omega_{B} - W_{BB}^{ + } (\Omega )}}, $$

Here, we adopt the relationship^44^

29 $$ \frac{{\omega^{2} }}{{c^{2} }}\sum\limits_{m} {\int {d^{3} {\mathbf{s}}\varepsilon_{I} ({\mathbf{s}},\omega )G_{nm} ({\mathbf{r}},{\mathbf{s}},\omega )G_{lm}^{*} ({\mathbf{r}}^{\prime},{\mathbf{s}},\omega )} } = {\text{Im}} [G_{nl} ({\mathbf{r}},{\mathbf{r}}^{\prime},\omega )], $$

and define $W_{ij}^{ + } (\Omega ) = \left\langle i \right.\left| {W^{ + } \left( \Omega \right)} \right|\left. j \right\rangle = \int_{0}^{{{ + }\infty }} {d\omega \frac{{\omega^{2} }}{{(\Omega + i\eta - \omega )\pi \varepsilon_{0} \hbar c^{2} }}{\mathbf{d}}_{i} \cdot {\text{Im}} [{\mathbf{G}}({\mathbf{r}}_{i} ,{\mathbf{r}}_{j} ,\omega )] \cdot {\mathbf{d}}_{j} }$ as coupling matrix element between state $\left| i \right\rangle$ and state $\left| j \right\rangle$. If $i = j$, it means local coupling. If $i \ne j$, it means radiative coupling. The detailed derivation of the results can be found from Eq. (30).

Combining Eqs. (27) and (28), we can obtain $c_{a}^{ + } (\Omega^{ + } )$ and $c_{b}^{ + } (\Omega^{ + } )$ in Eqs. (11) and (12). Similarly, by performing backward Fourier transform to Eqs. (5)–(7), we can obtain $c_{a}^{ - } (\Omega^{ - } )$ and $c_{b}^{ - } (\Omega^{ - } )$ in Eqs. (11) and (12).

### Coupling matrix element

The coupling matrix element can be derived as

30 $$ \begin{aligned} & \int_{0}^{{{ + }\infty }} {d\omega \frac{{\omega^{2} }}{{(\Omega \pm i\eta - \omega )\pi \varepsilon_{0} \hbar c^{2} }}{\mathbf{d}}_{i} \cdot {\text{Im}} [{\mathbf{G}}({\mathbf{r}}_{i} ,{\mathbf{r}}_{j} ,\omega )] \cdot {\mathbf{d}}_{j} } \\ & \quad = \mathcal{P}\int_{0}^{{{ + }\infty }} {d\omega \frac{1}{\Omega - \omega }\frac{{\omega^{2} }}{{\pi \varepsilon_{0} \hbar c^{2} }}{\mathbf{d}}_{i} \cdot {\text{Im}} [{\mathbf{G}}({\mathbf{r}}_{i} ,{\mathbf{r}}_{j} ,\omega )] \cdot {\mathbf{d}}_{j} } \\ & \quad \quad \mp i\int_{0}^{{{ + }\infty }} {d\omega \frac{{\omega^{2} }}{{\varepsilon_{0} \hbar c^{2} }}{\mathbf{d}}_{i} \cdot {\text{Im}} [{\mathbf{G}}({\mathbf{r}}_{i} ,{\mathbf{r}}_{j} ,\omega )] \cdot {\mathbf{d}}_{j} \delta (\Omega - \omega )} \\ & \quad = \frac{1}{2\pi }\mathcal{P}\int_{0}^{{{ + }\infty }} {d\omega \frac{{\Gamma_{ij} (\omega )}}{\Omega - \omega }} \mp i\frac{{\Omega^{2} }}{{\varepsilon_{0} \hbar c^{2} }}{\mathbf{d}}_{i} \cdot {\text{Im}} [{\mathbf{G}}({\mathbf{r}}_{i} ,{\mathbf{r}}_{j} ,\Omega )] \cdot {\mathbf{d}}_{j} \\ & \quad = \Delta_{ij} (\Omega ) \mp i\frac{{\Gamma_{ij} (\Omega )}}{2} \\ & \quad = W_{ij}^{ \pm } (\Omega ). \\ \end{aligned} $$

Here, $\mathcal{P}$ denotes the principal value integral, and we adopt the following definitions^26,51^

31 $$ \Gamma_{ij} (\Omega ) = \frac{{2\Omega^{2} }}{{\varepsilon_{0} \hbar c^{2} }}{\mathbf{d}}_{i} \cdot {\text{Im}} [{\mathbf{G}}({\mathbf{r}}_{i} ,{\mathbf{r}}_{j} ,\Omega )] \cdot {\mathbf{d}}_{j} , $$

32 $$ \Delta_{ij} (\Omega ) = \frac{1}{2\pi }\mathcal{P}\int_{0}^{{{ + }\infty }} {d\omega \frac{{\Gamma_{ij} (\omega )}}{\Omega - \omega }} . $$

$\Delta_{ij} (\Omega )$ can also be expressed as^32^

33 $$ \begin{aligned} \Delta_{ij} (\Omega ) & = \mathcal{P}\int_{0}^{{{ + }\infty }} {d\omega \frac{1}{\Omega - \omega }\frac{{\omega^{2} }}{{\pi \varepsilon_{0} \hbar c^{2} }}{\mathbf{d}}_{i} \cdot {\text{Im}} [{\mathbf{G}}({\mathbf{r}}_{i} ,{\mathbf{r}}_{j} ,\omega )] \cdot {\mathbf{d}}_{j} } \\ & = - \frac{{\Omega^{2} }}{{\varepsilon_{0} \hbar c^{2} }}{\mathbf{d}}_{i} \cdot {\text{Re}} [{\mathbf{G}}({\mathbf{r}}_{i} ,{\mathbf{r}}_{j} ,\Omega )] \cdot {\mathbf{d}}_{j} . \\ \end{aligned} $$

Regarding Eq. (33), Eq. (30) can be further expressed as

34 $$ \begin{aligned} W_{ij}^{ \pm } (\Omega ) & = - \frac{{\Omega^{2} }}{{\varepsilon_{0} \hbar c^{2} }}{\mathbf{d}}_{i} \cdot {\text{Re}} [{\mathbf{G}}({\mathbf{r}}_{i} ,{\mathbf{r}}_{j} ,\Omega )] \cdot {\mathbf{d}}_{j} \\ & \quad \mp i\frac{{\Omega^{2} }}{{\varepsilon_{0} \hbar c^{2} }}{\mathbf{d}}_{i} \cdot {\text{Im}} [{\mathbf{G}}({\mathbf{r}}_{i} ,{\mathbf{r}}_{j} ,\Omega )] \cdot {\mathbf{d}}_{j} \\ & = - \frac{{\Omega^{2} }}{{\varepsilon_{0} \hbar c^{2} }}{\mathbf{d}}_{i} \cdot {\mathbf{G}}^{/*} ({\mathbf{r}}_{i} ,{\mathbf{r}}_{j} ,\Omega ) \cdot {\mathbf{d}}_{j} . \\ \end{aligned} $$

Here, ${\mathbf{G}}^{/*}$ denote ${\mathbf{G}}$ and ${\mathbf{G}}^{*}$, corresponding to $W_{ij}^{ + }$ and $W_{ij}^{ - }$, respectively. Combining Eqs. (30) and (34), we can derive Eq. (13).

## Data Availability

The data that support the findings of this study are available from the corresponding author upon reasonable request.

## Code availability

The codes that support the findings of this study are available from the corresponding author upon reasonable request.
